# Transforming Growth Factor-β1/Smad7 in Intestinal Immunity, Inflammation, and Cancer

**DOI:** 10.3389/fimmu.2018.01407

**Published:** 2018-06-20

**Authors:** Edoardo Troncone, Irene Marafini, Carmine Stolfi, Giovanni Monteleone

**Affiliations:** Department of Systems Medicine, University of Rome Tor Vergata, Rome, Italy

**Keywords:** mucosal immunity, cytokines, inflammatory bowel diseases, colon cancer, antisense oligonucleotides

## Abstract

In physiological conditions, the activity of the intestinal immune system is tightly regulated to prevent tissue-damaging reactions directed against components of the luminal flora. Various factors contribute to maintain immune homeostasis and diminished production and/or function of such molecules trigger and/or propagate detrimental signals, which can eventually lead to chronic colitis and colon cancer. One such a molecule is transforming growth factor-β1 (TGF-β1), a cytokine produced by many inflammatory and non-inflammatory cells and targeting virtually all the intestinal mucosal cell types, with the down-stream effect of activating intracellular Smad2/3 proteins and suppressing immune reactions. In patients with inflammatory bowel diseases (IBD), there is defective TGF-β1/Smad signaling due to high Smad7, an inhibitor of TGF-β1 activity. Indeed, knockdown of Smad7 with a specific antisense oligonucleotide restores endogenous TGF-β1 activity, thereby inhibiting inflammatory pathways in patients with IBD and colitic mice. Consistently, mice over-expressing Smad7 in T cells develop severe intestinal inflammation in various experimental models. Smad7 expression is also upregulated in colon cancer cells, in which such a protein controls positively intracellular pathways that sustain neoplastic cell growth and survival. We here review the role of TGF-β1 and Smad7 in intestinal immunity, inflammation, and cancer.

## Introduction

**Figure 1:**
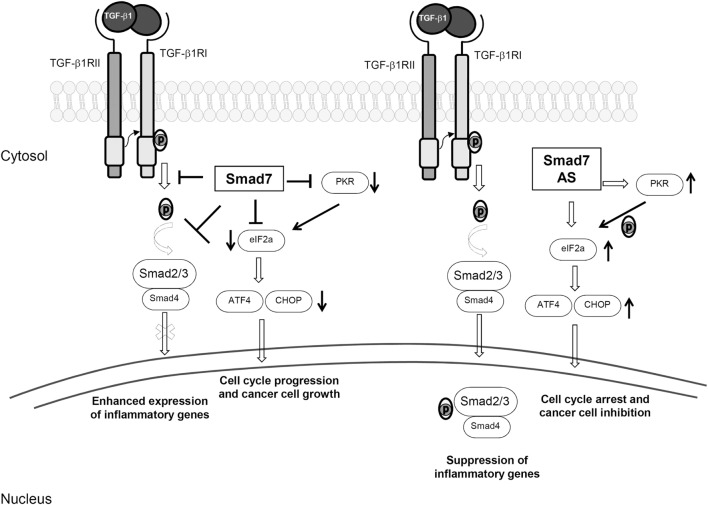
Smad7-induced biological effects. The left side of the figure shows the inhibitory effect of Smad7 on transforming growth factor (TGF)-β1 intracellular signaling. Smad7 binds to TGF-β receptor type I and prevents TGF-β1-driven Smad2/3 phosphorylation (p), thus sustaining inflammatory gene expression. High Smad7 prevents also eukaryotic translation initiation factor-2α (eIF2α) phosphorylation, either directly or through the inhibition of protein kinase RNA (PKR), thereby leading to downregulation of transcription factor 4 (ATF4) and CCAAT/enhancer binding protein homology protein (CHOP) with the down-stream effect of stimulating cell cycle progression and cancer cell growth. The right side of the figure shows the effect of Smad7 inhibition with a specific antisense oligonucleotide (AS). Smad7 inhibition restores TGF-β1-induced Smad2/3 phosphorylation, subsequent interaction of these two proteins with Smad4 and translocation of Smad2/3/4 complex to the nucleus, thereby suppressing inflammatory gene expression. Moreover, Smad7 knockdown causes TGF-β1-independent eIF2α phosphorylation, upregulation of ATF4 and CHOP with the down-stream effect of cell cycle arrest.

The gastrointestinal tract harbors a large number of commensal bacterial, viral, and fungal species, which trigger maturation of the mucosal immune system. There are over 300 Peyer’s patches and more than 30,000 isolated lymphoid follicles in the small and large intestines, and the lamina propria compartment is infiltrated with many CD4+ and CD8+ T cells, B cells, plasma cells, dendritic cells (DCs), and macrophages; T cells and various subsets of innate lymphoid cells (ILCs) are also present in the gut epithelium (1, 2). This state of “physiological inflammation” contributes to provide resistance to invading pathogens, while preserving barrier integrity and allowing normal absorptive and digestive functions. The maintenance of gut integrity and intestinal homeostasis depends also on the barrier effect of the epithelium, which limits translocation of luminal antigens and promotes immune regulation (3). Elegant studies in animal models of inflammation and observations in patients with chronic colitis strongly support this notion. Indeed, defects in epithelial barrier and/or lack of expression/function of counter-regulatory molecules can break the immune tolerance toward the luminal flora thus resulting in colitis and colorectal cancer (CRC) (2, 4–9). Data emerging from such studies indicate clearly that transforming growth factor (TGF)-β1 is one of the key molecules involved in the regulation of the epithelial cell biology and immunity in the gut (10, 11). TGF-β1 is a member of the TGF-β superfamily, which includes also TGF-β2, TGF-β3, bone morphogenetic proteins, and several growth and differentiation factors (12). In the gut, many immune and non-immune cells produce TGF-β1 and almost all the mucosal cells are targeted by this cytokine. TGF-β1 is secreted as part of a latent complex, which comprises latency-associated peptide (LAP) and latent TGF-β binding protein. In this form, TGF-β1 cannot bind to its receptor (13). TGF-β1 can be activated upon being released from the complex due to the proteolytic action of a number of proteinases or upon the interaction between the tripeptide integrin-binding motif on LAP and the correspondent binding sequence on αvβ3, αvβ5, αvβ6, or αvβ8 integrins expressed on the surface of epithelial cells, myofibroblasts, and DCs (14, 15). TGF-β1 signals through two transmembrane receptors with serine/threonine kinase activity, named TGF-β1 type 1 receptor (TβR1) and TGF-β1 type 2 receptor (TβR2) (13). Binding of TGF-β1 to TβR2 promotes auto-phosphorylation of the receptor and subsequent recruitment of TβR1 to form a transmembrane heterodimer. Then, the kinase activity of TβR2 determines phosphorylation of the regulatory glycine/serine-rich domain of TβR1 and, hence, the activated TβR1–TβR2 complex promotes phosphorylation of Smad2 and Smad3. Phosphorylated Smad2/3 proteins heterodimerize with Smad4 to generate a complex that moves to the nucleus, where it regulates the expression of target genes (13, 16) (Figure 1). TGF-β1-associated Smad signaling is a tightly controlled phenomenon, and another intracellular Smad protein, termed Smad7, acts as a negative regulator of such a pathway through various mechanisms. Smad7 can bind to TβR1 and compete with Smad2/3 for the catalytic site of phosphorylation, thus preventing the phosphorylation of Smad2/3 (Figure 1) (17, 18). Smad7 can recruit phosphatases to TβR1 thereby promoting de-phosphorylation and inactivation of the site (19) and can promote ubiquitination-driven proteasome-mediated degradation of TβR1 in association with E3 ubiquitin ligases SMURF1/2 (20, 21). Moreover, Smad7 can localize into the nucleus and inhibit the association of Smad2-3/Smad4 complex with target genes (22). Besides its inhibitory effect on TGFβ1 signaling, Smad7 regulates the expression and function of several molecules involved in the control of inflammation and carcinogenesis in a TGF-β1-independent manner (Figure 1).

We here review the role of TGF-β1 and Smad7 in intestinal immunity, inflammation, and cancer.

## TGF-β1 and Intestinal Homeostasis

**Figure 2:**
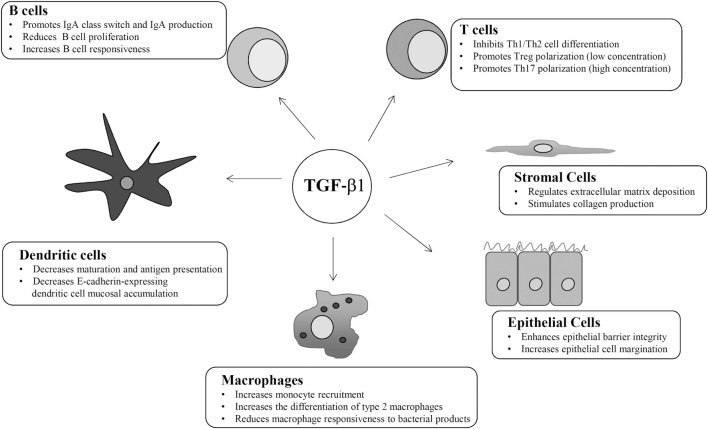
Schematic view of the main cell targets and biological function of transforming growth factor (TGF)-β1 in the gut.

TGF-β1 regulates the function of many mucosal cell types in an autocrine and paracrine manner (Figure 2). For instance, TGF-β1 suppresses proliferation and activation of CD4+ T helper (Th) lymphocytes (23). Mice with T-cell targeted deletion of TβR2 or transgenic mice expressing a dominant-negative of TβR2 are unable to respond to TGF-β1 and show a phenotype characterized by systemic autoimmunity and severe colitis (11). In both strains, activated T cells accumulate in multiple organs, including the gut, highlighting the role of TGF-β1 in blocking T cell activation, and maintaining intestinal immune tolerance. CD4+ T lymphocytes have a high grade of plasticity and can differentiate in various subsets depending on the specific cytokine milieu at the induction and effector sites (24). TGF-β1 strongly inhibits Th1 and Th2 differentiation. TGF-β1-induced inhibition of Th1 cell differentiation is mediated by a direct downregulation of the transcription factor Tbet, a master regulator of Th1 cell polarization (25). Moreover, TGF-β1 downregulates the expression of IL-12Rβ2 and prevents the expansion of Th1 responses driven by IL-12, a key cytokine in the induction of Th1-type immunity in humans (26). TGF-β1 can also directly downregulate GATA3, a transcription factor involved in Th2 cell differentiation (27, 28). In contrast, TGF-β1 promotes directly and indirectly T cells polarization toward a regulatory (T regulatory cells, Tregs) phenotype (29). Tregs express the transcription factor Foxp3 and exert regulatory functions by acting mainly on effector T cells (30). Mice with loss of TGF-β1 signaling have reduced numbers of circulating CD4+Foxp3+ Tregs, raising the possibility that TGF-β1 contributes to intestinal immune homeostasis in part by inducing Tregs differentiation (31, 32). This hypothesis is supported by studies demonstrating that TGF-β1 promotes generation of naturally occurring Tregs, a subset of Tregs, which are generated in the thymus early after birth, as well as differentiation of peripherally induced Tregs from naïve T cells (33–35). Interestingly, the number of CD4+Foxp3+ Tregs remains unaltered in mice lacking TGF-β1 in CD4+ T-cells, while TGF-β1-null mice have reduced numbers of Tregs. These findings suggest that peripheral differentiation of Tregs depends on TGF-β1 produced by other cell types rather than T cells. In the gut, CD103-expressing DCs are a major source of TGF-β1. These cells produce also elevated levels of retinoic acid (RA), which potentiates TGF-β1-induced expansion of Tregs because of a direct effect on Foxp3 promoter (36, 37). TGF-β1 and RA promote the *in vitro* differentiation of naïve T cells in another group of Foxp3-expressing Tregs, termed induced Tregs (38). TGF-β1 plays also a role in the interaction between intestinal immune system and gut microbiota. *Clostridium butyricum* promotes Tregs generation through the induction of TGF-β1 by colonic lamina propria DCs (39).

TGF-β1, along with IL-6, IL-21, and IL-1β, contributes also to the differentiation of Th17 cells, a CD4+ T cell subset characterized by the expression and activity of the master regulator retinoid acid-related orphan receptor-γt and producing various cytokines, including IL-17A (40). It has also been demonstrated that commitment of naïve CD4+ T cells along the Th17 or Tregs phenotype depends on TGF-β1 concentration, given that low concentration of the cytokine promotes Tregs differentiation through downregulation of IL-23 receptor, while high concentration of TGF-β1, in conjunction with IL-6 and IL-21, upregulates IL-23 receptor and promotes Th17 polarization (41).

TGF-β1 controls memory CD8+ T cells. Mice with deletion of TβR2 show a reduced number of antigen-specific memory CD8+ T cells in the gut, and this phenomenon seems to be, at least in part, secondary to a reduced expression of integrins (42).

TGF-β1 is also a relevant regulator of B cell and plasm-cell biology. TGF-β1 in B cells mediates IgA class-switch and promotes IgA production (43, 44). Mice lacking TGF-β signaling in B cells do not develop intestinal inflammation, but deletion of TβR2 in CD19-expressing B cells associates with B cell hyperplasia in Peyer’s patches, modifications in B cell responsiveness and serum IgA deficiency (44, 45). Secretory IgAs control the bacterial composition, as they neutralize luminal bacteria by enhancing phagocytosis and improving the ability of DCs to present antigens. Moreover, IgAs block surface epitopes of luminal bacteria, thus inhibiting bacterial adhesion to the intestinal epithelium (46). In Peyer’s patches, B cells and DCs interact through TGF-β1-activated integrin αvβ8, and this interaction promotes IgA production (47). TGF-β1 regulates IgA production through the canonical Smad-mediated pathway as the absence of Smad2 results in IgA deficiency, while overexpression of Smad3 and 4 determines an increased IgA production (48, 49).

Innate lymphoid cells are a family of hematopoietic cells involved in host defense, immune homeostasis, and tissue remodeling. These cells belong to the innate immune system and are abundantly present at mucosal sites where they act as a first line of defense against pathogens (50). Although there is no clear evidence that TGF-β1 regulates the function of ILCs in the gut, it is known that TGF-β1 guides the differentiation of type 1 ILCs in salivary glands through a Smad4-independent pathway (51).

TGF-β1 is involved in the control of intestinal DC function. Mice with deletion of TGF-β signaling in DCs due to a selective lack of TβR2 develop systemic autoimmunity and colitis, the later being characterized by loss of goblet cells and marked mucosal lymphocytic infiltration with altered Tregs differentiation, T cells and B cells with an activated phenotype, and increased expression of pro-inflammatory cytokines (52, 53). As mentioned above, DCs produce TGF-β1 and at the same time contribute to TGF-β1 activation. Mice lacking integrin β8 in DCs fail to activate TGF-β1 and spontaneously develop colitis (54). TGF-β1 negatively regulates the mucosal accumulation of other DC subtypes, such as those expressing the adhesion molecule, E-cadherin. Interestingly, adoptive transfer of E-cadherin-expressing bone marrow DCs into T-cell-restored Rag1-deficient mice enhances Th17 cell responses and exacerbates colitis (55).

TGF-β1 is an important regulator of macrophage function. TGF-β1 produced by intestinal epithelial cells can act as a chemokine and stimulate recruitment of blood monocytes to the intestinal mucosa (56). Moreover, TGF-β1 promotes differentiation of type 2 macrophages, a subset of anti-inflammatory cells, and reduces the macrophage responsiveness to bacterial products, thus promoting an anergic state that is crucial to maintain intestinal homeostasis (57). Mice with selective knockout of TβR2 in macrophages do not show spontaneous inflammation in the gut, but develop a more severe colitis after DSS administration, with reduced levels of IL-10, further confirming the ability of the cytokine to trigger anti-inflammatory signals in macrophages (58).

TGF-β1 targets also non-immune cells, including epithelial cells and stromal cells. Both these cell types produce elevated amounts of the cytokine. TGF-β1 promotes the expression of tight junction protein (i.e., Claudin-1) and adhesion molecules, thus enhancing epithelial barrier integrity, and is a powerful inducer of intestinal epithelial cell margination, a phenomenon that facilitates wound healing (Figure 2) (59). Mice with selective deletion of TGFβ1 signaling in the intestinal epithelium do not develop inflammation but are more susceptible to DSS-colitis (60). TGF-β1 stimulates stromal cells (i.e., myofibroblasts) to produce collagen and is a crucial regulator of extra-cellular matrix deposition (Figure 2), a phenomenon that is relevant for wound healing processes (61). A poorly controlled TGF-β1-induced extra-cellular matrix deposition has been involved in the development of intestinal strictures, such as those complicating Crohn’s disease (CD) natural history (62, 63). In the gut, fibrogenesis is a complex and multifactorial process that involves several mediators, and TGF-β1 is supposed to be the most relevant pro-fibrogenic cytokine (64). TGF-β1 promotes fibronectin, type I collagen, and connective tissue growth factor production in fibroblasts isolated from CD strictures and enhances fibroblasts contractile activity (65).

## TGF-β/Smad7 in Intestinal Inflammation

CD and ulcerative colitis (UC), are chronic, relapsing inflammatory disorders of the gastrointestinal tract and represent the two most relevant forms of IBD in humans (66). The etiology of IBD is unknown but accumulating evidence suggests that IBD are multifactorial diseases in which environmental and genetic factors trigger an abnormal immune response against component of intestinal microflora (67). In both disorders, defects of counter-regulatory factors/mechanisms contribute to amplify mucosal inflammatory signals. One such defect involves the TGF-β1/Smad pathway. In the intestine of healthy individuals, TGF-β1 functions are properly suggested by the constitutive elevated levels of phosphorylated Smad3. Moreover, *in vitro* treatment of normal intestinal mucosal samples with an anti-TGF-β1 antibody increases expression of pro-inflammatory molecules such as T-bet and IFN-γ and stimulation of normal lamina propria mononuclear cells (LPMC) with exogenous TGF-β1 inhibits NF-kB activity and reduces IL-8 production (68–70). In contrast, stimulation of IBD LPMC with TGF-β1 neither inhibits NF-kB activation nor reduces the production of pro-inflammatory mediators, highlighting the possibility that IBD cells are resistant to TGF-β1-mediated immune suppression (70). This is consistent with the demonstration that in inflamed intestine of IBD patients, there is reduced Smad2/3 phosphorylation (69). This finding associates with enhanced expression of Smad7 (69). Interestingly, analysis of Smad7 content in mucosal samples of IBD patients revealed that Smad7 is upregulated at protein but not RNA level, suggesting a post-transcriptional regulation of Smad7 (71). Indeed, we have previously shown that, in both CD and UC mucosa, Smad7 protein stability is sustained by post-translational mechanisms, which enhance Smad7 acetylation thereby inhibiting ubiquitination-driven proteasomal-mediated degradation. Such modifications are partly due to p300, as silencing of this transcription coactivator, which is over-produced in CD mucosa, reduces Smad7 acetylation thereby stimulating ubiquitination-driven proteasomal-mediated degradation (71). Additional factors are supposed to stabilize Smad7 expression in IBD. In this context, our data indicate that, in IBD tissue, cells over-expressing Smad7 have reduced levels of SIRT1, a component of the mammalian Sirtuin family proteins that deacetylates the lysine residues of Smad7 with the down-stream effect of reducing Smad7 expression (72).

## The Pathogenic Role of Smad7 in the Gut

Various approaches have been used to assess the role of Smad7 in IBD. Initially, IBD LPMC and mucosal explants were treated with a specific Smad7 antisense oligonucleotide (AS). Inhibition of Smad7 associated with enhanced Smad3 phosphorylation and reduced production of inflammatory cytokines (Figure 1) (69). Pre-incubation of IBD LPMC with a blocking TGF-β1 antibody abrogated the Smad7 AS-mediated effects, indicating that the anti-inflammatory function of Smad7 AS is mediated by TGF-β1. As pointed out above, TGF-β1 promotes differentiation of Foxp3-expressing Tregs and the activity of the cytokine is required for the function of such cells (73, 74). Mucosal IBD CD4+ T cells show resistance to Foxp3-expressing Tregs-mediated suppression and this has been associated with Smad7, as knockdown of Smad7 restores the responsiveness of effector CD4+ T cells to Foxp3-expressing Tregs (75). Like IBD patients, mice with trinitrobenzene sulfonic acid (TNBS-) and oxazolone-mediated colitis, two mouse models of colitis, which resemble CD and UC, respectively, express elevated levels of TGF-β1 in the inflamed colons (76, 77). In both these models, Smad7 expression is upregulated and p-Smad3 is reduced. Colitic mice given oral Smad7 AS exhibit enhanced p-Smad3 expression, reduced expression of inflammatory cytokines and a less severe colitis (78). We generated a T cell-specific Smad7 transgenic (Tg) mouse on C57B6 genetic background, which does not develop spontaneously colitis (75). However, using the T cell-transfer model of colitis, we showed that adoptive transfer of Smad7 Tg naïve CD4+ T cells into immunodeficient mice produced a more severe intestinal inflammation than that documented in mice reconstituted with wild-type cells, and colitis induced by Smad7 Tg cells was only partly inhibited by co-transfer of Tregs (75). Finally, we showed that Smad7 Tg mice develop a more severe colitis in comparison to wild-type mice after DSS administration (79).

T cells of Smad7 transgenic mice have reduced levels of aryl hydrocarbon receptor (AhR), a transcription factor that stimulates IL-22 production and promotes regulatory mechanisms in the gut (80). In the T-cell transfer colitis model, AhR activation significantly ameliorates the course of colitis driven by wild-type T cells, but does not influence colitis induced by Smad7 transgenic T cells. Consistently, in normal but not in IBD LPMC, TGF-β1 enhances AhR expression (81). Altogether, these data show an inverse correlation between AhR and Smad7 expression in the gut.

## Therapeutic Benefit of Smad7 Inhibition in Patients with Inflammatory Bowel Diseases

The demonstration that Smad7 is upregulated in IBD mucosa and inhibition of this protein allows endogenous TGF-β1 to dampen the ongoing mucosal inflammation paved the way for the development of an oral Smad7 AS-containing pharmaceutical compound. This drug, initially named GED0301 and later on mongersen, was formulated in order to facilitate the deliver of the active molecule in the terminal ileum and right colon, which are the primarily affected sites in CD (82). A phase 1, open label, dose-escalating clinical trial conducted in 15 patients with active, steroid-dependent/resistant CD showed that 7-day treatment was safe and well-tolerated by the patients and associated with clinical benefit (82). Patients enrolled in the trial were monitored for the development of intestinal strictures. Six months after the end of the treatment, no patient developed strictures (83). To further assess the impact of Smad7 inhibition on the development of intestinal fibrosis, we used a mouse model of TNBS-mediated colitis-driven intestinal fibrosis. Interestingly, treatment of colitic mice with mongersen reduced the degree of intestinal inflammation and limited the development of intestinal fibrosis (84).

A subsequent phase 2, multicenter double-blind, placebo-controlled clinical trial was conducted in steroid-resistant/dependent CD patients with inflammatory lesions confined to the terminal ileum and/or right colon. The study confirmed the safety profile of mongersen and showed that 55 and 65% of the patients treated with the highest doses of the drug (i.e., 40 or 160 mg/day for 2 weeks) achieved clinical remission (primary end-point) as compared to 10% in the placebo group (85). Responders to mongersen exhibited reduction in the serum levels of CCL20, a chemokine that contributes to recruit immune cells to the intestine ad is over-produced in the epithelium of CD patients (86). Next, an exploratory, phase 2, multicenter study confirmed the clinical efficacy of the drug and documented an endoscopic improvement in nearly one-third of the patients treated with mongersen for 4–12 weeks (87). Next, a phase III clinical trial was conducted in steroid-resistant/dependent CD patients with inflammatory lesions of the terminal ileum and/or colon and endoscopic evidence of active inflammation. The trial was discontinued in October 2017 as an interim analysis documented a lack of efficacy of the drug.

## SMAD7 in CRC

Colorectal cancer represents a leading cause of cancer-related morbidity and mortality, with 1.65 million new cases and almost 835,000 deaths estimated worldwide in 2015 (88). In 70% of cases, CRC arises as sporadic disease, with several environmental and genetic factors involved in the pathogenesis, most of which are still unknown (89). Instead, in 2% of cases, CRC arises in patients with long-standing UC or extensive CD (colitis-associated cancer, CAC), with a cumulative risk that has been related with disease duration, extension, and severity of inflammation (90, 91). TGF-β1 seems to play both pro-tumorigenic and anti-tumorigenic roles in CRC depending on the tumor stage and probably reflecting the complexity of TGF-β1 function and the large number of biological processes in which the cytokine is involved. While at early stages of tumorigenesis, TGF-β1 contributes to maintain cell differentiation and restricts epithelial cell growth, thus acting as a tumor suppressor, at later stages, it promotes epithelial–mesenchymal transition, neo-angiogenesis, cancer progression, and metastasis (12, 92). Similarly, a dual role of Smad7 has been described in various types of cancer with pro-tumorigenic or anti-tumorigenic effects according to the cancer site and biology (93). Single nucleotide polymorphisms of Smad7 gene associate with CRC (i.e., rs4939827, rs12953717) (93, 94). Moreover, in a study from Boulay and colleagues, Smad7 mutations were analyzed in 264 CRC biopsies, and amplification of Smad7 gene was associated with a poor prognosis, with a possible dose effect, while deletion of Smad7 gene associated with a better outcome (95). Our studies have recently evidenced a link between Smad7 expression in immune cells and CAC. In particular, a reduced number of Smad7-expressing CD4+ T lymphocytes were documented in the colonic mucosa of IBD patients who developed CAC compared to IBD patients with uncomplicated disease (79). In line with this observation, in an experimental model of CAC, Tg mice that overexpress Smad7 in T cells developed a more severe intestinal inflammation, characterized by an abundant infiltrate of cytotoxic CD8+ T cells and NKT cells, compared to control mice, and were largely protected from tumors. The anti-tumorigenic effect of Smad7 over-expression in T cells appeared related to the action of IFN-γ, as genetic deletion of such a cytokine abolished the protective effect of Smad7 on colon carcinogenesis (79). Consistent with these observations is the demonstration that Smad7 Tg mice develop less tumors than wild-type littermates following the subcutaneous injection of syngenic MC38 colon carcinoma cells (96). Altogether, these data suggest that high Smad7 in immune cells promotes the amplification of Th1-cytokine responses, which protect against colon carcinogenesis. However, in line with the above-referenced genetic studies, a different scenario emerges when the role of Smad7 is analyzed in sporadic CRC. Indeed, CRC cells produce huge amounts of Smad7 and knockdown of Smad7 with a specific AS inhibits the *in vitro* and *in vivo* growth of CRC cells (97). This effect of Smad7 AS relies on the modulation of cell cycle-related proteins and ultimately results in S phase arrest and cell death. Since these findings are seen in CRC cells unresponsive to TGFβ1 and the anti-proliferative effect of Smad7 AS is not affected by stimulation of CRC cells with TGF-β1 or anti-TGF-β1, it is highly likely that Smad7 exerts pro-tumorigenic effects in a TGFβ1-independent manner (Figure 1) (97). Analysis of the basic mechanisms underlying the mitogenic effect of Smad7 in CRC cells showed that Smad7 knockdown causes phosphorylation of eukaryotic translation initiation factor 2α (eIF2α), a transcription factor that regulates cell cycle arrest, and consequent upregulation of activating transcription factor 4 (ATF4) and CCAAT/enhancer binding protein homology protein (CHOP) (98). Silencing of the serine–threonine protein kinase RNA abrogates Smad7 AS induced eIF2α phosphorylation and ATF4/CHOP induction, thus preventing cell death (Figure 1) (98). In contrast, an anti-tumorigenic role for Smad7 has been recently reported by Wang and colleagues, who showed that nuclear reporter subfamily 2, group F, and member 2 (NR2F2), a molecule involved in many cancers, induces a TGFβ1-dependent epithelial–mesenchymal transition by inhibiting Smad7, thus promoting CRC metastasizing process (99).

Altogether, these data highlight the complex role of Smad7/TGFβ1 signaling in colon carcinogenesis.

## Conclusion

The findings discussed in this review underline the crucial role of TGF-β1 in the maintenance of intestinal homeostasis and suggest that defective function of this cytokine can contribute to trigger and/or amplify detrimental signals in the gut. There is also discussion on the expression of Smad7 in patients with IBD and the crucial role played by this protein in inhibiting TGF-β1 function and sustaining IBD-related inflammation. Knockdown of Smad7 allows endogenous TGF-β to suppress effector responses. In line with this, CD patients treated with oral compound containing Smad7 AS showed clinical and endoscopic improvement during phase 1 and phase 2 clinical trials. However, a recent phase 3 clinical trial has been discontinued apparently due to the lack of efficacy of the drug, but the reasons for the discrepancy between phase 2 and phase 3 studies remain unknown. Similarly, some issues regarding the expression/function of Smad7 in IBD and CRC remain to be addressed. For instance, we still do not known whether, in IBD, Smad7 is regulated in a cell-specific manner and which factors/mechanisms contribute to maintain the elevated levels of Smad7. It would also be relevant to know whether molecular profiling of IBD patients can help to identify better candidates to treatment with Smad7 inhibitors. Further experimentation is also needed to clarify whether and which regulatory effects of Smad7 on the ongoing mucosal inflammation and colon carcinogenesis are in part independent on TGF-β1, as it is known that Smad7 controls some biological functions in a TGF-β1-independent manner.

## Author Contributions

ET and IM wrote the manuscript. CS supervised parts of the project. GM designed the paper, supervised the project, and wrote the manuscript.

## Conflict of Interest Statement

GM has filed a patent related to the treatment of inflammatory bowel diseases with Smad7 antisense oligonucleotides, while the remaining authors have no conflict of interest.
